# Does Using None-of-the-Above (NOTA) Hurt Students’ Confidence?

**DOI:** 10.3390/jintelligence11080157

**Published:** 2023-08-07

**Authors:** Jeri L. Little

**Affiliations:** Department of Psychology, California State University, East Bay, 25800 Carlos Bee Blvd., Hayward, CA 94542, USA; jeri.little@csueastbay.edu

**Keywords:** multiple-choice, testing, assessment, learning, metacognition, confidence, none-of-the-above

## Abstract

Students claim that multiple-choice questions can be tricky, particularly those with competitive incorrect choices or choices like none-of-the-above (NOTA). Additionally, assessment researchers suggest that using NOTA is problematic for assessment. In experiments conducted online (with trivia questions) and in the classroom (with course-related questions), I investigated the effects of including NOTA as a multiple-choice choice alternative on students’ confidence and performance. In four experiments, participants answered two types of questions: basic multiple-choice questions (basic condition) and equivalent questions in which one incorrect choice was replaced with NOTA (NOTA condition). Immediately after answering each question, participants rated their confidence in their answer to that question (item-by-item confidence). At the end of the experiments, participants made aggregate confidence judgments for the two types of questions and provided additional comments about the use of NOTA as an alternative. Surprisingly, I found no significant differences in item-by-item confidence or performance between the two conditions in any of the experiments. However, across all four experiments, when making aggregate judgments, participants provided lower confidence estimates in the NOTA condition than in the basic condition. Although people often report that NOTA questions hurt their confidence, the present results suggest that they might not—at least not on a question-by-question basis.

## 1. Introduction

Tests containing multiple-choice questions are commonly used in educational contexts. In the present paper, “multiple-choice questions” refer to questions that include a prompt or stem and several possible alternatives, one of which is the correct answer (these are sometimes called “single-choice” items or “n-alternative-forced-choice” items, where “n” is the number of alternatives). Multiple-choice questions sometimes contain none-of-the-above (NOTA) as the last multiple-choice alternative. Students often report disliking NOTA as a choice, even when it is not the correct answer. Anecdotally, instructors hear things like, “it makes it a trick question” and “it makes me second guess myself.” Whether using NOTA as an incorrect choice actually hurts students’ confidence when they are taking a test is not a question that has been systematically examined. The main goal of the present experiments was to examine whether replacing an incorrect alternative with NOTA would affect participants’ confidence on an item-by-item basis—that is, whether NOTA would affect confidence even when it was not correct. I was also interested in participants’ ideas about NOTA in general and whether theories about NOTA would align with item-by-item confidence.

### 1.1. Multiple-Choice Testing: The Pros and Cons of Using NOTA

Multiple-choice questions are commonly used for assessment. They offer faster and more objective grading than other more open-ended questions (Butler 2018). Best practices for writing good multiple-choice questions (i.e., those that assess learning well) have been offered. Butler (2018) provided tips for educators like keeping the format simple and the questions targeted, aiming for moderately difficult questions, and—critically—avoiding none-of-the-above. Avoiding NOTA is a majority opinion among assessment researchers (e.g., Haladyna et al. 2002). DiBattista et al. (2014) found that using NOTA increased difficulty and reduced discriminability. Some assessment researchers take a weaker stance on NOTA. For example, in a large-scale study across 20 college-level tests, Frary (1991) assessed difficulty and discriminability and found that compared to the average non-NOTA item, the average NOTA item was slightly more difficult but about equal in discriminability. He concluded that contrary to typical assessment recommendations, NOTA does not need to be eliminated, but caution should be used when using it. 

In addition to being used for assessment, tests can be used as a tool to help students learn (e.g., practice tests, polling questions, review games). If information is tested, it is more likely to be retained than if it is simply restudied, a finding called the testing effect (e.g., Roediger and Karpicke 2006). Multiple-choice questions are often used as a tool for learning (see, e.g., Bjork et al. 2015; Marsh et al. 2007). 

Some research has examined how multiple-choice questions with NOTA influence retention of the tested content (Blendermann et al. 2020; Odegard and Koen 2007). When NOTA is the correct answer for a five-alternative multiple-choice practice question, students’ performance is often hurt as compared to basic four-alternative questions—both on the practice multiple-choice test and on later cued-recall tests; this is because students avoid choosing NOTA, consequently choosing an incorrect answer, and are then likely to provide an incorrect answer to a more open-ended questions later (e.g., Odegard and Koen 2007). In subsequent research, Blendermann et al. (2020) had participants answer three types of trivia questions: cued-recall, multiple-choice with NOTA in which NOTA was correct, and multiple-choice with NOTA in which NOTA was not correct (note that participants did not answer basic multiple-choice questions in this study). Additionally, half of the participants were prompted to recall the answer if they chose NOTA. Because items were counterbalanced across conditions (i.e., all participants saw the same prompts), the researchers were able to show that participants answered more questions correctly when they were presented in a cued-recall format than when they were presented as an MC-NOTA question when NOTA was correct. That is, participants avoided NOTA even if they could have answered the question correctly in a cued-recall format (Blendermann et al. 2020).^1^

But NOTA on practice tests need not be universally problematic. In order to understand why NOTA could be a useful alternative, especially on a practice question for learning, it is important to consider other research on multiple-choice testing. Multiple-choice questions are good for learning when they include plausible alternatives because plausible alternatives introduce competition that induces test-takers to think not only about why a given choice is correct but also about why other choices are wrong (Little and Bjork 2015; Little et al. 2019). Such broadened retrieval processes can improve recall for related information later (Little et al. 2012, 2019; Little and Bjork 2015). In theory, adding NOTA could introduce additional competition that could be beneficial for learning. That is, the “second guessing” that students often report may mean that these students are exerting more effort thinking about their answer, which may be good for learning (c.f., Bjork and Bjork 2020). As such, when included in multiple-choice practice questions, NOTA could have benefits that might outweigh their costs. Although Odegard and Koen (2007) found that using NOTA as the correct alternative hurt performance, they also found that using NOTA as an incorrect alternative did not hurt performance, suggesting that NOTA may only be problematic when it is the correct response. Additionally, following from the research on how multiple-choice testing influences the retention of related information by Little and colleagues, Blendermann et al. (2020) found that compared to practice cued-recall questions, practice multiple-choice questions that included NOTA improved later recall of a previously incorrect alternative as the correct answer to a related question; overall, it did not matter whether NOTA was correct or incorrect on the initial practice test to see this effect (the correct answer to a related question was always an incorrect alternative on the initial multiple-choice test). 

### 1.2. Metacognition

Metacognition reflects participants’ beliefs about their learning. Confidence ratings reflect participants’ beliefs about the accuracy of their current answer. People sometimes have accurate metacognition (i.e., metacognitive ratings that reflect their performance), but sometimes they do not. Why is metacognition sometimes inaccurate? Ackerman (2019) and others argue that people cannot “read” the quality of their cognition directly, but must instead rely on various cues drawn from the task, environment, or their subjective experience (see also Dunlosky and Tauber 2014; Koriat 1997). Sometimes, the cues are misleading. For example, people often believe they have learned less on tasks that required more effort, “reading” high effort as indicative of weaker knowledge or a likelihood of performing poorly later (e.g., Benjamin et al. 1998). Bjork and Bjork (2011, 2020) termed “desirable difficulties” for situations (and their findings) in which conditions of practice that are more effortful (difficult) for the learner lead to better learning; interestingly, learners often predict worse learning for conditions that require more effort (e.g., spacing rather than massing to-be-learned information, (Kornell and Bjork 2008); testing rather than restudying, (Roediger and Karpicke 2006)). Consistent with the idea that learners perceive effort as indicative of weaker knowledge, longer response times to answer a question are associated with reduced confidence (e.g., Ackerman and Zalmanov 2012; Hertwig et al. 2008). If people believe that they need to use more effort to answer questions with NOTA, that increased effort would be expected to be associated with reduced confidence. 

Aspects of the task that affect metacognitive judgments can be whether a manipulation occurs between subjects/lists versus within subjects/lists. Within-lists manipulations are usually more sensitive to differences in judgments because these manipulations induce direct comparisons between conditions (Begg et al. 1989; Shaw and Craik 1989; see also, Tullis et al. 2013). 

Metacognitive judgments can also be influenced by when they are made (Ackerman 2019). Metacognitive assessments sometimes come after a given item (i.e., item-by-item judgments), and sometimes they come after a set of items (i.e., global, aggregate judgments). These different judgments may be made based on different cues. Item-by-item judgments tend to focus participants more on the item, but global/aggregate judgments tend to focus participants on broader theories about learning (e.g., see Koriat et al. 1980, 2004; Koriat 2008a). Item-focus may promote experience-based judgments (e.g., based on fluency or perceived speed of retrieval) or information-based judgments (e.g., ideas about knowledge of the item itself; Koriat 2008a). Basing judgments on broader theories of learning is also more likely in a within-subjects manipulation than in a between-subjects manipulation (Koriat et al. 2004). Item-by-item judgments tend to be more accurate (and more overconfident) than aggregate judgments (see, e.g., Mazzoni and Nelson 1995; Sniezek and Buckley 1991). However, even when participants are focused on the item, aspects of the item that do not influence later recall can affect metacognitive judgments (e.g., font size, Rhodes and Castel 2008).

### 1.3. The Present Experiments

In the present experiments, I examined confidence ratings and performance for basic multiple-choice questions with competitive alternatives as compared to questions for which one of those alternatives was replaced with none-of-the-above. Assessing confidence—both item-by-item and aggregate confidence—was the main goal of the present research; however, whether confidence is accurate or not depends on its relationship to performance, so I also investigated performance. Although Odegard and Koen (2007) showed that adding NOTA as an alternative reduced performance, they compared a five-alternative NOTA condition to a four-alternative basic condition. In the current experiments, I examined performance for items in the NOTA condition as compared to those in a basic condition that has the same number of alternatives (i.e., NOTA replaced an alternative). Experiments 1a and 1b were conducted online using trivia questions (E1a used college students; E1b used participants from MTurk). Experiments 2a and 2b were conducted in a cognitive psychology course with questions based on topics taught in that course. Based on anecdotal reports and the existing literature (e.g., Bjork and Bjork 2020), I predicted that questions with NOTA would reduce average confidence as compared to those same questions without NOTA. 

## 2. Experiment 1a

### 2.1. Method

#### 2.1.1. Participants

There were 48 participants from California State University, East Bay (14 males, 33 females, and 1 non-binary). Their ages ranged from 18 to 38 (*M* = 20.5, *SD* = 4.3). They participated as part of the subject pool requirement for a psychology course. No previous research existed to provide clear effect size estimates, so given the strength of beliefs regarding the use of NOTA among students and the literature, I used a small-medium to medium effect (*d* = 0.4–0.5) to estimate sample size. This required 34 to 52 participants, respectively, to achieve 80% power for paired-samples t-tests (G*Power 3.1.9.4). The motivation for sample sizes in subsequent experiments will be discussed in the General Discussion.

#### 2.1.2. Design

A within-subjects design was used, with item type (NOTA, basic) as the independent variable. I examined performance and confidence ratings for each question as well as aggregate confidence ratings at the end of the task. 

#### 2.1.3. Materials

Multiple-choice trivia questions (24 critical questions and 6 filler questions, to be described below) were modified from those used by Little (2018), and the materials are all available at OSF (DOI 10.17605/OSF.IO/N4A7K). They contained five competitive alternatives; in the basic condition, these alternatives were all provided. An example is: *What is the name for currency in Korea? a. yen, b. won, c. peso, d. renminbi, (yuan), e. rupee*, Answer: *won*. In the NOTA condition, the last alternative (e.g., rupee in the example above) was swapped out for “none of the above.” For the 24 critical items, when none-of-the-above was used, it was always incorrect (i.e., one of the competitive alternatives was always correct). So that NOTA would be correct 20% of the time that it was included, I created six filler items for which NOTA was correct (a given participant received three of these). 

#### 2.1.4. Procedure

Participants completed the experiment online using Qualtrics. After providing consent, they were told that they would be answering trivia questions and that after each question, they would have to rate their confidence from 10 (totally a guess) to 50 (know) in 10-point increments (i.e., 10, 20, 30, 40, or 50). They were told to avoid consulting any outside resources (e.g., internet or friends), even to just check spelling or even after they answered the question. They were told that even though it is tempting, looking up answers would compromise the integrity of the project. To check that they read the instructions, they were then asked “Can you look up answers or ask your friends?” After they responded, they were reminded that they could not look up answers until the experiment is over. 

**Figure 1:**
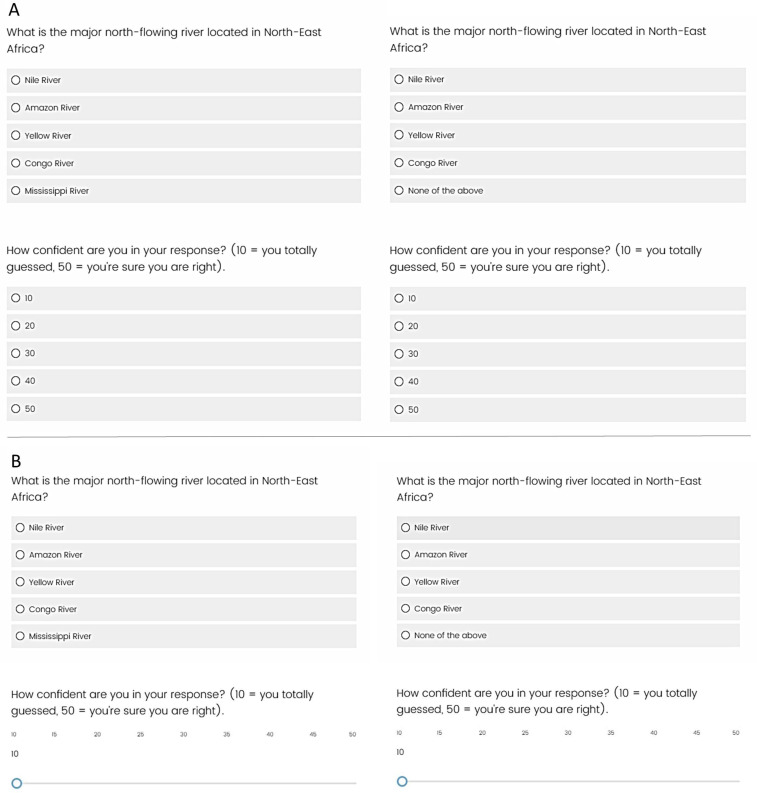
Examples of questions and confidence rating prompts in Experiments 1a and 1b. Panel (**A**) shows how an example question and confidence scale appeared on the screen in Experiment 1a when that item was in the basic condition (**left**) and NOTA condition (**right**). Panel (**B**) shows how an example question and confidence scale appeared on the screen in Experiment 1b when that item was in the basic condition (**left**) and NOTA condition (**right**).

They then answered 27 multiple-choice questions in a random order, with 12 of the questions being from the basic condition, 12 of the questions being from the NOTA condition (but NOTA was never correct), and 3 questions being filler items (NOTA was included and was the correct answer). A given question and its confidence rating were presented on the same screen, and timing was self-paced. No feedback was provided. Which items served in the basic condition and the NOTA condition were counterbalanced across participants, and participants were randomly assigned to one of the two counterbalanced conditions. See Panel A of Figure 1 for an example of a question and confidence estimate for both the basic and NOTA conditions, respectively.

Then, participants completed a 2 min visual search task as a distractor. After the distractor task, participants completed a cued-recall retention test with the previously tested items, except that the items did not contain any alternatives. Participants could spend up to 20 s per question, but they had to spend at least 4 s per question.

Finally, participants answered questions about the task. First, they were told that some of the questions had none-of-the-above as a choice and some did not and were asked whether they tended to have more confidence on questions “with none of the above,” questions “without none of the above,” or “confidence was the same for both types of question.” Then, participants provided their average confidence rating for the two conditions on a sliding scale from 10 to 50, where participants could use 1-point increments to slide the curser to an appropriate number (With NOTA and without NOTA were shown on the same screen). Finally, I asked participants an open-ended question about why they chose the response they chose. I also collected demographic information and information about their experience with the experiment (e.g., whether they had computer problems or consulted outside resources). 

### 2.2. Results 

Performance, item-by-item confidence, and aggregate confidence for the NOTA and basic conditions for all four experiments are shown in Table 1. Paired-comparisons were assessed with paired-samples *t* tests. Additionally, results of Bayesian analyses (BFs) are provided for all non-significant results in order to assess the evidence in favor of an absence of an effect. 

Seven participants were removed because they reported looking up answers. 

In this and all subsequent experiments, I compared the questions in the basic condition to the comparable questions in the NOTA condition for which NOTA was incorrect. Filler items for which NOTA was correct were included to avoid a test-taking strategy in which participants would ignore NOTA, but these were analyzed separately for descriptive purposes. Performance and confidence ratings for the filler items were not of primary interest and are included in Table 2. Data, including answers to open-ended questions, are available for all experiments at OSF (DOI 10.17605/OSF.IO/N4A7K).

As shown in Table 1, average correct performance on the multiple-choice questions in the NOTA condition (*M* = 53%, *SE* = 3%) was not different than performance in the basic condition (*M* = 56%, *SE* = 3%), *t*(40) = 0.63, *p* > .05, *d* = 0.10, 95% CI [−10%, 5%]. There was moderate evidence in favor of an absence of an effect of item type on performance (BF = 6.76). 

Additionally, as shown in Table 1, the average item-by-item confidence ratings were not different in the NOTA condition (*M* = 27.9, *SE* = 1.1) than in the basic condition (*M* = 27.8, *SE* = 1.1), *t*(40) = 0.06, *p* > .05, *d* = 0.01, 95% CI [−1.9, 2.0]. There was moderate evidence in favor of an absence of an effect of item type on confidence (BF = 8.20). 

Goodman–Kruskal gamma correlations were computed to assess the rank–order relationship between performance and confidence (relative metacognitive accuracy; see Nelson 1984). It is common in metamemory research to calculate gamma coefficients for each participant and then to calculate central tendency (means or medians) of those coefficients across participants to assess relative metacognitive accuracy (e.g., Dunlosky and Nelson 1992; Koriat 2008b, 2019; Nelson and Dunlosky 1991). Coefficients were calculated for each participant for the basic condition, NOTA condition, and for the combination of the two conditions (i.e., the critical items). Sometimes, gamma coefficients could not be calculated because a participant obtained the same performance for all items (i.e., all correct or all incorrect) or provided the same confidence rating for all items in the set. Missing values in the basic or NOTA conditions would necessitate removing participants from subsequent analyses. In order to maintain gamma correlations for all participants, I replaced the missing gamma correlations with 0 (c.f., Serra and Shanks 2023). Average gamma correlations for the 24 critical items (*M* = 0.67, *SE* = 0.03) was significantly greater than zero, *t*(40) = 19.91, *p* < .001, *d* = 3.11, 95% CI [0.60, 0.74]; an analysis of the difference in median gamma coefficients compared to zero showed the same outcome, *p* < .05, by a one-sample Wilcoxon Signed-Rank Test. Average gamma correlations were not different in the NOTA condition (*M* = 0.69, *SE* = 0.05) as compared to the basic condition (*M* = 0.66, *SE* = 0.04), *t*(40) = 0.59, *p* > .05, *d* = 0.09, 95% CI [−0.09, 0.16]; an analysis of the difference in median gamma coefficients showed same outcome, *p* > .05, by a related-samples Wilcoxon Signed-Rank Test.

Performance on the final cued-recall test was not different in the NOTA condition (*M* = 41%, *SE* = 3%) as compared to the basic condition (*M* = 42%, *SE* = 3%), *t*(40) = 0.36, *p* > .05, *d* = 0.06, 95% CI [−8%, 6%]. There was moderate evidence in favor of an absence of an effect of item type on performance (BF = 7.72).

However, as shown in Table 1, when participants were asked to provide ratings at the end of the experiment, they provided significantly lower confidence ratings for the NOTA condition (*M* = 26.5, *SE* = 1.5) than for the basic condition (*M* = 31.7, *SE* = 1.7), *t*(37) = 2.79, *p* < .05, *d* = 0.45, 95% CI [−8.9, −1.4]^2^. Additionally, when given a forced-choice question about preference, 12% said they prefer items with NOTA, 68% said they prefer items without NOTA, and 20% said that it did not make a difference. 

Participants also answered a free-recall question about why they preferred one choice over another. Several of the responses—in line with the ideas put forth in the introduction to this paper—indicated that NOTA shakes their confidence. Responses that align with this idea included “By adding none-of-the-above[,] it means that there might be more answers[,] and it tends to make me overthink my original answer,” “it makes you feel like something is wrong and doubt yourself,” and “none-of-the-above questions are trickier.” However, there were participants who said that “it does not change anything for [them].” Participants that said they preferred answers with NOTA often said things that suggested that they did not understand the question (“because the answer is there within the options provided, and it helps by remembering the answer”), or they based their answer on test-taking strategies (that may or may not be accurate). For example, one participant said, “When I do not know, [none of the above is] my choice. Sometimes teachers only put it when it is the correct answer”.

### 2.3. Discussion

Contrary to my expectations, I did not obtain any difference for item-by-item confidence ratings between the basic and NOTA conditions (when NOTA was incorrect). However, consistent with my expectations, participants said that they preferred questions without NOTA, and when asked to provide overall ratings for confidence for the two conditions (aggregate confidence), they provided significantly lower ratings for the NOTA condition than for the basic condition.

One possible reason for the dissociation between item-by-item performance and aggregate performance is that participants used different cues for these two judgments. Koriat et al. (2008) distinguished between information-based judgments, experience-based judgments, and theory-based judgments. Information-based judgments depend on the strength of retrieved domain-specific information. Experience-based judgments rely on the online processing of items, which may include feelings of fluency, effort, or perceived time to respond. Theory-based judgments depend upon general metacognitive beliefs, participants’ own competence, and/or how various factors can affect performance. Theory-based judgments are especially likely to be used in conditions in which the manipulation is within-subjects and aggregate judgments are made (Koriat et al. 2004). In the present experiment, it may be the case that participants relied on information-based processes for judgments for the item-by-item confidence despite the within-subject manipulation but theory-based judgments for the aggregate judgments. Because the NOTA questions and basic questions were actually similar in difficulty (as demonstrated by performance on those questions), the information-based judgments were—in this case—more accurate, at least in terms of absolute accuracy. Relative accuracy of confidence ratings was very good in the present study. Average gamma correlations were quite high, indicating that participants’ confidence reflected their knowledge. Further, gamma correlations did not differ between the two conditions, providing evidence that replacing a competitive alternative with NOTA did not affect one’s ability to make accurate confidence judgments.

One could be concerned that the lower aggregate confidence estimates in the NOTA versus basic condition was affected by the filler items (i.e., items for which NOTA was correct). This is unlikely to be the case, however. As indicated in Table 2, confidence was numerically higher for those filler items than for the items in the NOTA condition. 

There is a possibility that the scales for the aggregate judgments allowed for less precision than did the scales in the item-by-item judgments, and the lack of precision for the item-by-item judgments may have concealed differences that I would otherwise observe. Experiment 1b used the same materials and procedure as Experiment 1a, with one exception: I used sliders with 1-point increments for both item-by-item ratings and aggregate ratings (still starting at 10 and ending at 50). 

Although a secondary issue, it is worth mentioning that I also did not find a difference between the two conditions on performance: either on the initial multiple-choice tests or on the final cued-recall test. On the one hand, this is consistent with the idea that when NOTA serves as an incorrect alternative, it does not hurt the testing effect (Blendermann et al. 2020). On the other hand, not finding an advantage for NOTA items is inconsistent with the idea that NOTA provides a “desirable difficulty” (c.f., Bjork and Bjork 2020). However, research on desirable difficulties tends to show more pronounced advantages with longer rather than shorter delays. I will discuss this issue further in the General Discussion.

## 3. Experiment 1b

### 3.1. Method

#### 3.1.1. Participants

Fifty-nine participants were recruited from Mturk. There were 38 males and 21 females and their ages ranged from 20 to 67 (*M* = 36.9, *SD* = 10.6). They received USD 3 for their participation. 

#### 3.1.2. Design, Materials, and Procedure

The design, materials, and procedure were the same as those in Experiment 1a with one exception. I used sliders with 1-point increments for both item-by-item ratings. See Panel B of Figure 1 for an example of the question and confidence judgment. 

### 3.2. Results 

Nine participants were removed because they reported looking up answers, or their answers were suspicious (text appeared to be copied and pasted from the internet). As in Experiment 1a, performance on the multiple-choice test, item-by-item confidence, and aggregate confidence are shown in Table 1. 

As in Experiment 1a, average correct performance on the multiple-choice questions in the NOTA condition (*M* = 64%, *SE* = 3%) was not different than performance in the basic condition (*M* = 68%, *SE* = 3%), *t*(49) = 1.72, *p* > .05, *d* = 0.24, 95% CI [−10%, 1%]. There was low evidence in favor of an absence of an effect of item type on performance (BF = 2.2). 

Additionally, and also consistent with the results of Experiment 1a, the average confidence ratings provided after each item was not different in the NOTA condition (*M* = 32.0, *SE* = 1.1) than in the basic condition (*M* = 33.0, *SE* = 1.0), *t*(49) = 1.57, *p* > .05, *d* = 0.22, 95% CI [−2.4, 0.3]. There was low evidence in favor of an absence of an effect of item type on confidence (BF = 2.8). 

Average gamma correlations for the 24 critical items (*M* = 0.63, *SE* = 0.04) was significantly greater than zero, *t*(49) = 16.04, *p* < .001, *d* = 2.27, 95% CI [0.55, 0.71]; an analysis of the difference in median gamma coefficients compared to zero showed the same outcome, *p* < .05, by a one-sample Wilcoxon Signed-Rank Test. Average gamma correlations were not different in the NOTA condition (*M* = 0.60, *SE* = 0.05) as compared to the basic condition (*M* = 0.52, *SE* = 0.06), *t*(49) = 0.96, *p* > .05, *d* = 0.14, 95% CI [−0.08, 0.22]; an analysis of the difference in median gamma coefficients showed the same outcome, *p* > .05, by a related-samples Wilcoxon Signed-Rank Test.

Again consistent with the results of Experiment 1a, performance on the final test was not different in the NOTA condition (*M* = 57%, *SE* = 3%) as compared to the basic condition (*M* = 60%, *SE* = 3%), *t*(49) = 0.86, *p* > .05, *d* = 0.12, 95% CI [−8%, 3%]. There was moderate evidence in favor of an absence of an effect of item type on performance (BF = 6.3).

And finally, also consistent with the results of Experiment 1a, when participants were asked to provide ratings at the end of the experiment, they provided significantly lower confidence ratings for the NOTA condition (*M* = 28.7, *SE* = 1.3) than for the basic condition (*M* = 33.6, *SE* = 1.3), *t*(49) = 4.07, *p* < .05, *d* = 0.58, 95% CI [−7.3, −2.5]. When given a forced-choice question about preference, 3% said they prefer items with NOTA, 22% said they prefer items without NOTA, and 50% said that it did not make a difference. 

Participants also answered a free-recall question about why they preferred one choice over another. As in Experiment 1a, several of the responses indicated that NOTA shakes their confidence. Responses that align with this idea included that they preferred questions without NOTA because for “without NOTA” questions “there is a sure answer within the options” and “if it has none of the above, it might be a trick.” Although very few participants said that they prefer questions with NOTA, in this experiment as compared with Experiment 1a, a larger percentage of participants said that question type did not matter. Free responses consistent with this idea included, “if I know the answer, it does not matter to me what option is available.”

### 3.3. Discussion

As in Experiment 1a, I did not obtain any differences for item-by-item performance nor for item-by-item confidence ratings between the basic and NOTA conditions (when NOTA was incorrect). Additionally, participants said that they preferred questions without NOTA (or it did not matter), and when asked to provide overall ratings for confidence for the two conditions, they provided significantly lower ratings for the NOTA condition than for the basic condition. 

The results of these first two experiments provide a disconnect between aggregate responses/open-ended responses and item-by-item responses. Null results should be taken with a grain of salt, but even collapsing over the data from the two experiments, there was no difference between the NOTA and basic conditions for performance, *t*(90) = 1.61, *p* > .05, *d* = 0.17, 95% CI [−8%, 1%] or average item-by-item confidence ratings, *t*(90) = 0.97, *p* > .05, *d* = 0.10, CI [−1.7, 0.6]. There was moderate evidence in favor of an absence of an effect of item type on performance (BF = 3.4) and confidence (BF = 7.6). 

When considering the educational application of these experiments, one may wonder whether this finding would replicate in a real classroom context. In Experiments 2a and 2b, I conducted a version of the experiment in a cognitive psychology course. Given that performance on a later cued-recall test also did not differ and my main goal was to examine confidence as it pertained to current performance, Experiments 2a and 2b did not use a final test.

## 4. Experiment 2a

### 4.1. Method

#### 4.1.1. Participants

There were 42 participants from the author’s cognitive psychology course at California State University, East Bay during Fall 2022. There were 14 males and 27 females, and their ages ranged from 20 to 46 (*M* = 24.1, *SD* = 5.6). Students needed to complete the experiment for classwork points (as a review exercise at the end of the semester), but they were told that they could obtain extra credit (5 points, out of 1000 possible points in the course) for consenting to have their data analyzed for the purposes of a research study. 

#### 4.1.2. Design

As in Experiments 1a and 1b, a within-subjects design was used, with item type (NOTA, basic) as the independent variable. I examined performance and confidence ratings for each question as well as aggregate confidence ratings at the end of the task. 

#### 4.1.3. Materials

I created 10 multiple-choice questions, each with 4 competitive alternatives. These questions covered a range of topics covered in the course (perception, memory, and judgment), and were similar in difficulty to questions I provide on quizzes and exams. Some were from or modified from the test bank from Reisberg’s *Cognition: The Science of the Mind* textbook (Hanson 2015). One example was: *Trina believes that firefighters are braver than police officers. She tends to notice firefighters who are brave and to discount police officers doing acts of bravery. Which of the following is likely at play? a. availability heuristic, b. confirmation bias, c. anchoring and adjustment heuristic, d. representativeness heuristic*; Answer: *confirmation bias*. As in the other experiments, in the NOTA condition, the last alternative was swapped out for “none of the above.” For the NOTA versions of the 10 critical items, none-of-the-above was always incorrect (i.e., one of the competitive alternatives (A–C) was always correct). I also included one filler NOTA item for which none-of-the-above was correct so that one of the six NOTA questions given participant would answer would be correct. This filler question was the same for all participants. The materials are available at OSF (DOI 10.17605/OSF.IO/N4A7K).

#### 4.1.4. Procedure

Participants completed the experiment as a review activity from their own devices, either in class or at home during the last week of the semester (students could participate in class or via Zoom). The instructor explained that the responses in the review activity would be used for research so long as students consented. They were told that they would need to complete the activity for their daily classwork points, but if they consented to have their results analyzed, they could earn 5 points of extra credit (out of 1000 total points in the course). 

Upon clicking on the link (via Qualtrics), they were provided with a consent form and instructions. They were told that they would answer multiple-choice questions and then would have to rate their confidence from 10 (totally a guess) to 50 (know) in 1-point increments. They then answered 11 multiple-choice questions in a predetermined order (consistent with the order in which the content was taught), with five of the questions being from the basic condition, five of the questions being from the NOTA condition, and one question being a filler item for which NOTA was correct. The questions were self-paced, and no feedback was provided. Which items served in the basic condition and the NOTA condition were counterbalanced across participants, and participants were randomly assigned to one of the two counterbalanced conditions. 

After answering the questions, participants were told that some of the questions had none-of-the-above as a choice and some did not, and were asked whether they tended to have more confidence on questions “with none of the above,” questions “without none of the above,” or “confidence was the same for both types of questions.” Then, participants provided their average confidence rating for the two conditions on a sliding scale from 10 to 50, where participants could use 1-point increments. Finally, participants were asked an open-ended question about why they chose the response they chose as well as whether they completed the activity before or after the instructor went over the answers in class (after everyone said they were finished).

Unlike in Experiments 1a and 1b, there was no distractor task nor a final cued-recall test.

### 4.2. Results

One participant was removed because they reported completing the study after the instructor reviewed the answers. 

Average correct performance on the multiple-choice questions in the NOTA condition (*M* = 68%, *SE* = 4%) was not different than performance in the basic condition (*M* = 68%, *SE* = 4%), *t*(40) = 0.00, *p* > .05, *d* = 0.00, 95% CI [−10%, 10%]. There was moderate evidence in favor of an absence of an effect of item type on performance (BF = 8.21). 

Additionally, the average of confidence ratings provided after each item were not different in the NOTA condition (*M* = 34.9, *SE* = 1.3) than in the basic condition (*M* = 34.3, *SE* = 1.2), *t*(40) = 0.49, *p* > .05, *d* = 0.08, 95% CI [−1.9, 3.1]. There was moderate evidence in favor of an absence of an effect of item type on confidence (*BF* = 7.32). 

Average gamma correlations for the ten critical items (*M* = 0.38, *SE* = 0.08) was significantly higher than zero, *t*(40) = 4.95, *p* < .001, *d* = 0.77, 95% CI [0.23, 0.53]; an analysis of the difference in median gamma coefficients compared to zero showed the same outcome, *p* < .05, by a one-sample Wilcoxon Signed-Rank Test. Average gamma correlations were not different in the NOTA condition (*M* = 0.34, *SE* = 0.10) as compared to the basic condition (*M* = 0.26, *SE* = 0.09), *t*(40) = 0.50, *p* > .05, *d* = 0.08, 95% CI [−0.24, 0.40]; an analysis of the difference in median gamma coefficients showed same outcome, *p* > .05, by a related-samples Wilcoxon Signed-Rank Test.

When participants were asked to provide ratings at the end of the experiment, they provided significantly lower confidence ratings for the NOTA condition (*M* = 26.7, *SE* = 1.6) than for the basic condition (*M* = 36.6, *SE* = 1.5), *t*(40) = 5.80, *p* < .01, *d* = 1.27, 95% CI [6.4, 13.3]. When given a forced-choice question about preference, 7% said they prefer items with NOTA, 68% said they prefer items without NOTA, and 24% said that it did not make a difference. 

Free responses were similar to those reported in the earlier experiments. One participant reported “When I see the option none of the above[,] it sometimes makes me second guess myself.” Someone else said, “I feel like it makes the question more tricky.” Someone reported, “When there is a none-of-the-above option, I begin to doubt myself even more because that makes me overthink the answer[,] even if it [is] right in front of me”. 

### 4.3. Discussion

The results of Experiment 2a replicated those of Experiments 1a and 1b and extended the findings to a real educational setting. Although this was a low-stakes “quiz” for participation points, the content was similar to content that would be tested on the final exam the following week. Thus, participants in this experiment were motivated (and likely more so than in Experiments 1a and 1b) to think about the questions and treat the activity like an assessment.

The accuracy of confidence ratings (as indicated by gamma correlations) was lower in this experiment than in the previous two experiments. Having fewer items (5 in each condition as compared to the 12 in each condition in Experiments 1a and 1b) made it more likely that participants would answer all questions correctly (or incorrectly) or provide the same confidence ratings for all items, which increased the number of participants who had gammas that could not be calculated. In those cases, gamma was replaced with 0, which reduced the average. Additionally, the questions in the present experiment may also have been harder to assess metacognitively than those used in the first two experiments, as the questions in the present experiments tended to be more conceptual (rather than factual) than those in Experiments 1a and 1b. Nevertheless, participants’ confidence was fairly accurate in terms of relative accuracy, and it did not differ across the two conditions.

## 5. Experiment 2b

### 5.1. Method

#### 5.1.1. Participants

There were 47 participants from the first author’s cognitive psychology course at California State University, East Bay during Spring 2023. There were 11 males and 36 females, and their ages ranged from 20 to 51 (*M* = 24.4, *SD* = 5.4). They participated for extra credit. 

#### 5.1.2. Design, Materials, and Procedure

The experiment was the same as Experiment 1b with one exception. One question used in Experiment 1a was confusing, and so it was modified. 

### 5.2. Results

Six participants were removed because they reported completing the study after listening to the answers. 

Replicating the results of the previous experiments, average correct performance on the multiple-choice questions in the NOTA condition (*M* = 66%, *SE* = 4%) was not different than performance in the basic condition (*M* = 66%, *SE* = 4%), *t*(40) = 0.00, *p* > .05, *d* = 0.00, 95% CI [−12%, 12%]. There was moderate evidence in favor of an absence of an effect of item type on performance (BF = 8.21). 

Similarly, the average of confidence ratings provided after each item was not different in the NOTA condition (*M* = 33.3, *SE* = 1.0) than in the basic condition (*M* = 33.2, *SE* = 1.0), *t*(40) = 0.14, *p* > .05, *d* = 0.02, 95% CI [−1.8, 2.1]. There was moderate evidence in favor of an absence of an effect of item type on confidence (BF = 8.13).

Average gamma correlations for the ten critical items (*M* = 0.40, *SE* = 0.07) was significantly higher than zero, *t*(40) = 5.82, *p* < .001, *d* = 0.91, 95% CI [0.26, 0.54]; an analysis of the difference in median gamma coefficients compared to zero showed the same outcome, *p* < .05, by a one-sample Wilcoxon Signed-Rank Test. Average gamma correlations were not different in the NOTA condition (*M* = 0.38, *SE* = 0.10) as compared to the basic condition (*M* = 0.19, *SE* = 0.09), *t*(40) = 1.33, *p* > .05, *d* = 0.21, 95% CI [−0.10, 0.47]; an analysis of the difference in median gamma coefficients showed same outcome, *p* > .05, by a related-samples Wilcoxon Signed-Rank Test.

Also replicating the earlier experiments, when participants were asked to provide ratings at the end of the experiment, they provided significantly lower confidence ratings for the NOTA condition (*M* = 26.9, *SE* = 1.4) than for the basic condition (*M* = 33.4, *SE* = 1.6), *t*(40) = 3.21, *p* < .05, *d* = 0.50, 95% CI [2.4, 10.5]. When given a forced-choice question about preference, 10% said they prefer items with NOTA, 68% said they prefer items without NOTA, and 22% said that it did not make a difference. 

Participants also answered a free-recall question about why they preferred one choice over another. Several of the responses indicated that NOTA shakes their confidence. Responses that align with this idea included “[NOTA] answers give reason to doubt a correct answer” and “I feel like this can be a trick question.” A student in this class also mentioned how NOTA affects elimination strategies: “Because having ‘without none of the above’ [questions] makes it easier [to] select a[n] answer and [use] process of elimination, [whereas] ‘with none of the above’ [questions] may throw people off in answering[,] causing people to think about the answers and choose that one.” Someone else said, “I feel like the option ‘none-of-the-above’ throws off my confidence when I select an answer.”

### 5.3. Discussion

Experiment 2b replicated the findings of the first three experiments. 

Taken together, collapsing across the data in Experiments 2a and 2b, there is strong evidence for a lack of an effect of item type on performance, *t*(81) = 0.00, *p* > .05, *d* = 0.00, 95% CI [−8%, 8%], BF = 11.48 and item-by-item confidence, *t*(81) = 0.48, *p* > .05, *d* = 0.05, 95% CI [−1.9 1.2], BF = 10.23. 

Similarly, as discussed for Experiments 1a and 1b, the filler item for which NOTA was correct was not included in the analysis of the NOTA condition. It is reasonable to assume that confidence for this item could have impacted participants’ aggregate judgments, and low confidence for this item could explain the lowered confidence for NOTA items in the aggregate ratings. This did not appear to be the case. In Experiments 2a and 2b, the question for which NOTA was correct had particularly alluring incorrect alternatives, so correct performance (i.e., correctly choosing NOTA) was very low for this item (22% in Exp. 2a and 7% in Exp. 2b). However, confidence for that item was comparable to confidence for the items in the NOTA condition (slightly higher in Exp. 2a, slightly lower in Exp. 2b). Thus, it is unlikely that this item is what led to the lower aggregate confidence ratings in the NOTA versus the basic condition. 

## 6. General Discussion

I tested the “common sense” belief that adding NOTA as an alternative hurts one’s confidence when answering test questions. Across four experiments, I failed to find evidence that replacing a competitive alternative with NOTA hurts item-by-item confidence or performance. This was surprising in light of the existing literature and anecdotal evidence which suggested that NOTA hurts assessment and confidence. However, consistent with anecdotal evidence, participants provided lower aggregate confidence estimates for questions with NOTA than for questions without NOTA. Additionally, answers to free-response questions often included ideas about participants second-guessing themselves and doubting an answer. In short, there is a disconnect between what educators, students, and even researchers think about how NOTA affects confidence and how it actually affects confidence (or even performance) on an item-by-item basis in situations in which NOTA replaces an otherwise competitive alternative. 

### 6.1. Metacognitive Beliefs about NOTA

The experiments revealed a discrepancy between what participants seem to believe about NOTA in general and their item-by-item confidence ratings. The aggregate judgments, responses to the multiple-choice metacognitive question about NOTA, and responses to the open-ended question tended to align with the common belief that NOTA makes questions harder and shakes one’s confidence. The item-by-item confidence ratings, by contrast, did not show that NOTA had a meaningful impact on confidence, and interestingly, this corresponded to participants’ actual performance. 

The discrepancy between the various global judgments and item-by-item judgments is consistent with research showing that aggregate (global) and item-by-item judgments can rely on different cues, with aggregate judgments being more likely to draw upon participants’ theories, and item-by-item judgments being more likely to draw upon aspects of the items themselves (Koriat et al. 2004). From the open-ended responses, in particular, it was clear that many participants were familiar with and had clear ideas about NOTA, so it is not surprising that those theories affected their aggregate judgments. Considering item-by-item judgments, confidence ratings were correlated with performance (gammas ranging from 0.38 to 0.67), indicating that a lack of a difference in average item-by-item judgments between the two conditions was not a result of confidence ratings that were made randomly. The strong correlations (particularly for Experiments 1a and 1b) also support the idea that the item-by-item judgments were based to a large extent on participants’ knowledge of the items themselves (or at least their beliefs about their knowledge in those domains), not their theories about NOTA. This is interesting in light of the idea that the within-subjects manipulation should have primed people to use theory-based cues even for the item-by-item judgments. However, it is consistent with the idea that the judgments were informed by information-driven processes (Koriat et al. 1980). One possible explanation is that these materials (trivia and information needed for a course) or perhaps the judgments themselves (confidence) promote more attention to individual items than would other lab-based materials (e.g., word-pairs) or metacognitive judgments (JOLs). Also interesting—and possibly worth future research—is the finding that the average gamma correlation was numerically higher in the NOTA condition than in the basic condition across all four experiments. 

### 6.2. Performance and the Implications of Using NOTA as a Desirable Difficulty

Performance in the NOTA and basic conditions was not different in any of the four experiments. This was interesting because assessment researchers have tended to find that questions with NOTA are associated with lower performance (Frary 1991). However, assessment research tends not to compare NOTA questions to basic questions that are the same question but with NOTA replaced by an additional competitive alternative. In the real world, questions that include NOTA may tend to be different from non-NOTA questions in other ways that affect performance and discriminability.

Would questions with NOTA always be equal in performance to comparable questions without NOTA? Probably not. First, if I had compared the basic condition to a NOTA condition that added NOTA as a choice (rather than as a replacement), performance would likely have been lower in the NOTA condition than in the basic condition (Odegard and Koen 2007). Although there are some advantages to this strategy (e.g., alternatives are consistent across conditions), the disadvantage is that choosing one of four choices correctly should—in general—be easier than choosing one of five choices correctly. I also did not randomly remove an alternative from the basic condition to create items in the NOTA condition. Sometimes, the alternative I removed was fairly competitive (e.g., representativeness heuristic in the example for Experiment 2a), but sometimes it was not as competitive (e.g., Mississippi River in the example in Figure 1). It is possible that had I removed different alternatives, the results would have been different. 

Students report that questions with NOTA make them “second guess themselves.” In the introduction, I argued that this second guessing might mean that they are using more effort, and this effort might be good for learning, a so-called “desirable difficulty” (c.f., Bjork and Bjork 2020). Based on this framework, I might have expected to see better performance on the final test in the NOTA condition than in the basic condition, but I did not. This may be because the delay between the initial and final test was very short. Benefits for the effortful condition tend to be more pronounced at longer delays, and sometimes, the benefit does not appear at a short delay at all. For example, Roediger and Karpicke (2006) found an advantage for testing over restudy, but only at a delay of 48 h or 1 week, not at a shorter 5 min delay. In Experiments 1a and 1b, the delay between initial test and later test was only 2 min. The classroom procedure used in Experiments 2a and 2b could have been used to look at the effect, but the final exam questions were not systematically related to the materials used in those experiments. To better examine whether NOTA could function as a desirable difficulty, future research should investigate whether using NOTA could have advantages as a tool for learning using delays that are educationally realistic.

If including NOTA as an alternative induces more effort, one would expect that answering those questions would take more time. The present experiments were not conducted with the idea that response times would be analyzed as a dependent variable. Timing was recorded in Experiments 1a and 1b, but a major limitation was that a given response time included both the time to answer that question and the time to answer the confidence prompt associated with that question. Additionally, because the studies were conducted online, the response times are not precise. With these limitations in mind, an analysis of the timing data for Experiments 1a and 1b failed to reveal that participants spent significantly longer time on questions and confidence ratings in the NOTA condition than on questions and confidence ratings in the basic condition. In fact, participants spent numerically more time on questions and confidence ratings in the basic condition. Perhaps if response time had differed, confidence would have as well (Ackerman and Zalmanov 2012). This finding would have been consistent with participants relying upon experience-based cues. Additionally, response time data—in combination with the results mentioned above—raise the question of whether adding NOTA, at least in these contexts, could actually act as a desirable difficulty. 

It is also worth noting that researchers have found that multiple-choice questions focused on reasoning tasks were less likely to lead participants to use valid cues for metacognition than were more open-ended tests (like cued-recall), owing to the belief that multiple-choice questions require less cognition processing (Wang et al. 2023; see also Ackerman 2019). Future research could compare cued-recall to various MC formats.

### 6.3. Additional Limitations and Future Directions

One concern is that the experiments were not sensitive enough to observe differences between the basic and NOTA conditions. This is unlikely because in all experiments, I did observe a difference between the NOTA and basic conditions in the aggregate judgments. Following Experiment 1a, I was concerned about the sensitivity of the scale of the item-by-item judgments, but Experiments 1b, 2a, and 2b all used scales in 1-point increments for item-by-item judgments. Additionally, each of these experiments utilized a within-subject manipulation, which should have been especially likely to promote differences in confidence between the two conditions (Begg et al. 1989; Shaw and Craik 1989). After Experiment 1a, I did not report typical power analyses. This is because the effect sizes obtained for confidence (average *d* = 0.08) were so low that sample size based on power would not have been reasonable (more than 1000 participants per study). Instead, I opted to report the results of Bayesian analyses as well. In most cases, there was moderate evidence for the null effect, and in the case of combining Experiments 2a and 2b, I found strong evidence for the null effect. At least with these materials and conditions, it is clear that any effect of replacing a competitive alternative with NOTA on item-by-item confidence is probably too small to be worth considering for practical purposes.

As mentioned earlier, participants were metacognitively accurate in their item-by-item confidence estimates in terms of comparing the two conditions to one another (by not estimating a difference). If performance differed, participants would be correct to provide different confidence estimates for item-by-item judgments (as well as for aggregate judgments). It would be interesting to test a scenario in which we knew performance would be higher in the NOTA condition and see whether that would change participants’ item-by-item and aggregate confidence. For example, if one were to replace a particularly attractive incorrect alternative with NOTA, participants might perform better in the NOTA condition. Imagine a question about the capital of Australia with the choices: Melbourne, Brisbane, Canberra, and Sydney. Because Sydney is a well-known city, it is a particularly attractive incorrect alternative. Thus, were one to replace Sydney with NOTA, participants might perform better on the NOTA version than on the basic version because (a) they would not be distracted by Sydney and (b) they would be more likely to carefully consider the alternatives and correctly choose Canberra as the answer. 

Another question of interest is why I chose a 10–50 scale rather than a more common 0–100% scale. First, choosing 0% would not make sense given that a guess on a five-alternative question should warrant 20% confidence. Thus, I could have used a 20% to 100% scale (or 25% to 100% for the experiments with four-alternative questions), but reasoning in this way can be unintuitive to participants. I opted to use 10–50 to separate the judgment from percentages, and participants could use this scale to predict performance. This scale, however, makes it difficult to assess the absolute accuracy of the confidence judgments. Future research could examine whether the type of confidence scales would affect metacognitive outcomes. 

What about all-of-the-above (AOTA)? People often consider none-of-the-above and all-of-the-above questions together. The processes used in these two questions are quite different, however. When one chooses AOTA, they are endorsing the other choices; they are not having to endorse an item that is not present or a range of possible items that are not present. There is inherently less ambiguity. Additionally, AOTA questions are often easier because if students know that more than one alternative is right, they can choose AOTA without knowing whether the others are correct (Butler 2018). Of course, questions can get even more complicated (e.g., A and B are correct; A, B, and C are correct). However, even with these types of items, the answer is always present. Future research could examine confidence with AOTA as an alternative. Although AOTA tends to make questions easier, participants may not be sensitive to that fact unless they have been taught it as a test-taking strategy.

### 6.4. Applications and Conclusion 

What should educators do with the knowledge gained from this set of experiments? There are still valid reasons to avoid NOTA. Butler (2018) suggested that NOTA does not serve as an ideal assessment because choosing a non-answer is not showing support for an answer. Odegard and Koen (2007) and Blendermann et al. (2020) found evidence that people tend to avoid choosing NOTA. Taken together, these ideas suggest that educators should avoid NOTA as a correct answer. However, perhaps the use of NOTA should not be universally discarded, as there are reasons that it can be worthwhile—maybe especially when used in practice or low-stakes multiple-choice test conditions. The addition of NOTA does not appear to impact participants’ confidence on an item-by-item basis, nor to hurt their performance in a significant way. Although the idea that using NOTA might serve as a desirable difficulty is not supported by the present experiments, the use of NOTA appears not to impair learning either. As discussed, when NOTA is wrong (as was the case in the items at the focus of these studies), there is little to no cost on previously tested information, and regardless of whether it is right or wrong, testing with NOTA can also help retention of related information (Blendermann et al. 2020). It is important to consider that when using NOTA in this manner (i.e., it is never correct), there is the risk that students will adopt a test-taking strategy that will detract from assessment, so one would need to occasionally use questions for which NOTA is correct. Used sparingly, however, and mostly on practice assessments, students are unlikely to develop that strategy. When NOTA has to be correct, one can use it on a question for which participants are likely to be confident in their knowledge of the answer. As one participant in Experiment 1b said, “if I know the answer, it does not matter to me what option is available.” 

In summary, although many educators, assessment researchers, and students condemn NOTA as being likely to hurt performance and/or confidence, the present research fails to show evidence for this idea, at least for item-by-item judgments and performance.

## Figures and Tables

**Table 1:** Mean Performance and Confidence for NOTA and Basic Multiple-choice Questions.

	Performance		Item-by-Item Confidence		Aggregate Confidence	
	NOTA	Basic	NOTA	Basic	NOTA	Basic
Exp. 1a	53% (3%)	56% (3%)	27.9 (1.1)	27.8 (1.1)	26.5 (1.5)	31.7 (1.7)
Exp. 1b	64% (3%)	68% (3%)	32.0 (1.1)	33.0 (1.0)	28.7 (1.3)	33.6 (1.3)
Exp. 2a	68% (4%)	68% (4%)	34.9 (1.3)	34.3 (1.2)	26.7 (1.6)	36.6 (1.5)
Exp. 2b	66% (4%)	66% (4%)	33.3 (1.0)	33.2 (1.0)	26.9 (1.4)	33.4 (1.6)

Note. Standard errors are in parentheses.

**Table 2:** Mean Performance and Confidence for the Filler Questions.

	Performance	Item-by-Item Confidence
Exp. 1a	48% (5%)	29.5 (1.6)
Exp. 1b	59% (4%)	35.7 (1.3)
Exp. 2a	22% (7%)	37.8 (1.8)
Exp. 2b	7% (4%)	31.2 (1.9)

Note. These data represent the filler items for which NOTA was correct. Standard errors are in parentheses.

## Data Availability

Materials and data can be downloaded at OSF: (https://doi.org/10.17605/OSF.IO/N4A7K).

## Notes

1 Because Blendermann et al. were interested in comparing NOTA (answer correct) to NOTA (answer incorrect) questions, the number of these two types of items was equal in their design. Although a valid experimental design decision for their purposes, having equal numbers of questions for which NOTA is and is not correct is not educationally realistic because NOTA correct answers are overrepresented; this design feature may be a reason that participants avoided choosing NOTA (Odegard and Koen 2007 made a similar design decision).

2 Four participants failed to provide aggregate ratings.
